# Supplementary figures and images for: Structural and molecular insight into the pH-induced low-permeability of the voltage-gated potassium channel Kv1.2 through dewetting of the water cavity
Source: PLoS Comput Biol. 2020 Apr 21;16(4):e1007405. doi: 10.1371/journal.pcbi.1007405 (PMC7173763; doi:10.1371/journal.pcbi.1007405)

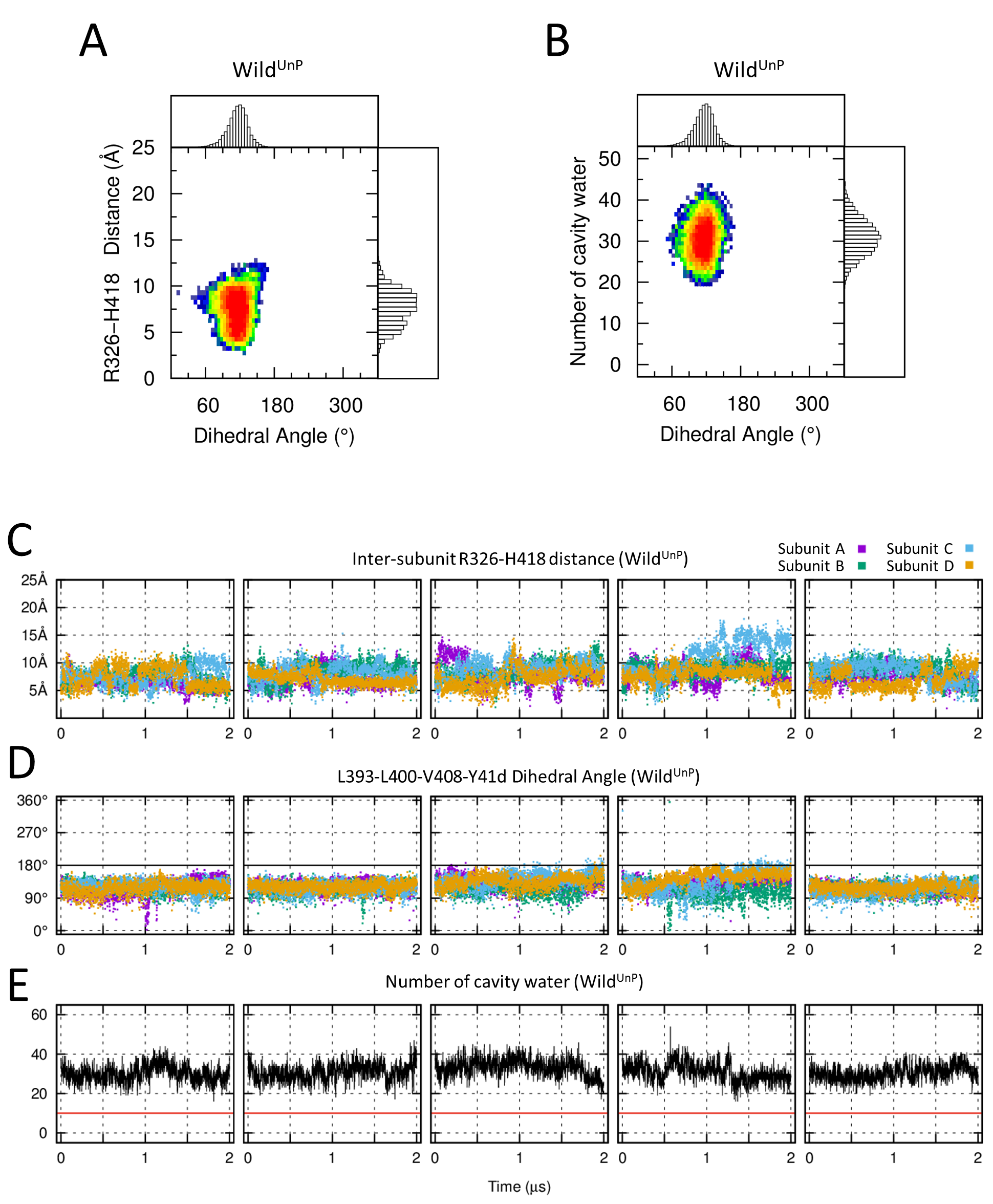

Supplement: S1 Fig — (A) Ensembles population of log for the R326–H418 distances and dihedral angles, and (B) ensembles population of log scale for the dihedral angles and number of water molecules in the water-filled cavity. The time evolution of (C) the distance between R326 and H418 residues, (D) the dihedral angle, and (E) the number of water molecules in the water cavity from five individual trajectories for WildUnP. (TIF) [file pcbi.1007405.s001.tif]

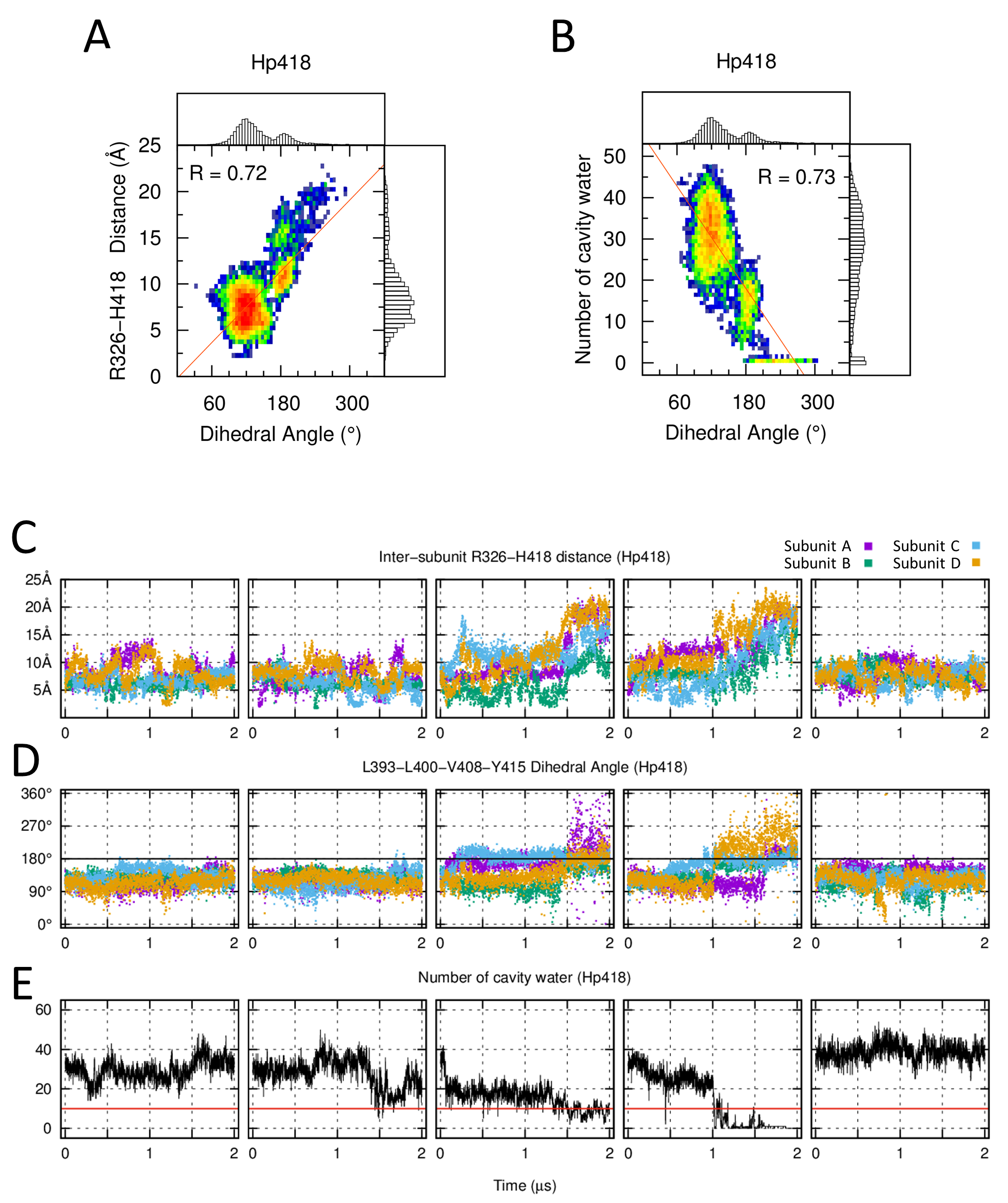

Supplement: S2 Fig — (A) Ensembles population of log for the R326–H418 distances and dihedral angles, and (B) ensembles population of log scale for the dihedral angles and number of water molecules in the water-filled cavity. The time evolution of (C) the distance between R326 and H418 residues, (D) the dihedral angle, and (E) the number of water molecules in the water cavity from five individual trajectories for Hp418. (TIF) [file pcbi.1007405.s002.tif]

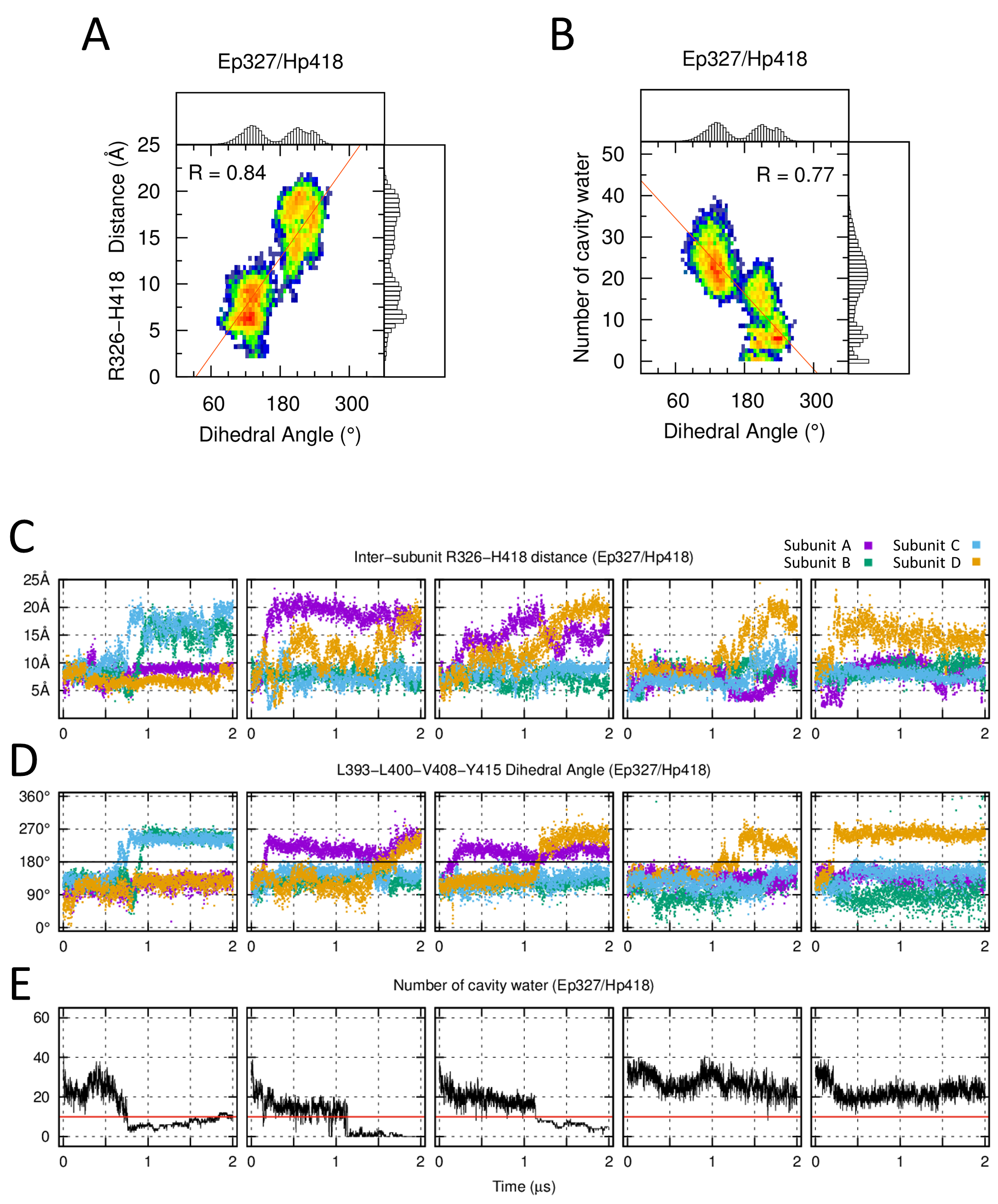

Supplement: S3 Fig — (A) Ensembles population of log for the R326–H418 distances and dihedral angles, and (B) ensembles population of log scale for the dihedral angles and number of water molecules in the water-filled cavity. The time evolution of (C) the distance between R326 and H418 residues, (D) the dihedral angle, and (E) the number of water molecules in the water cavity from five individual trajectories for Ep327/Hp418. (TIF) [file pcbi.1007405.s003.tif]

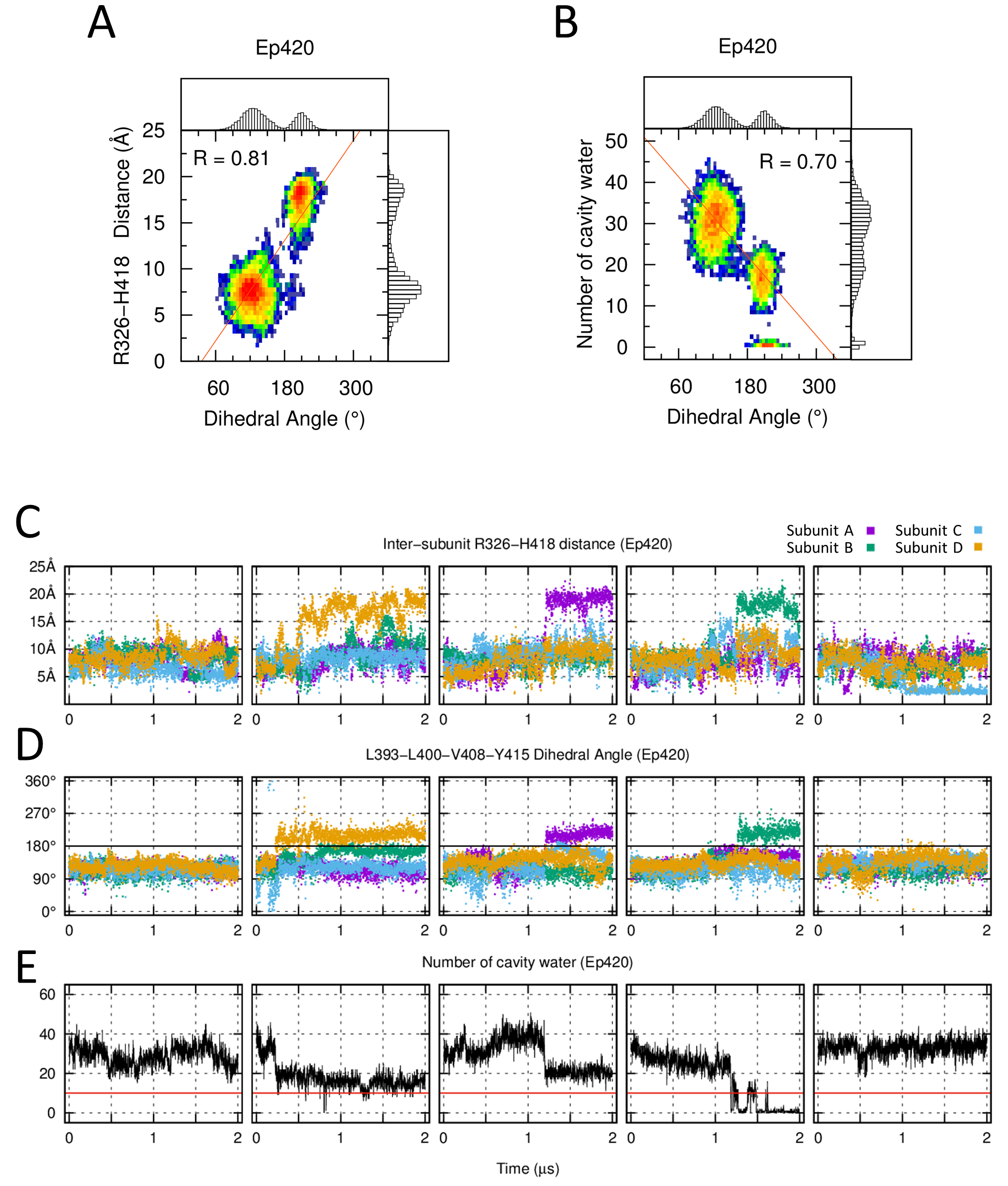

Supplement: S4 Fig — (A) Ensembles population of log for the R326–H418 distances and dihedral angles, and (B) ensembles population of log scale for the dihedral angles and number of water molecules in the water-filled cavity. The time evolution of (C) the distance between R326 and H418 residues, (D) the dihedral angle, and (E) the number of water molecules in the water cavity from five individual trajectories for Ep420. (TIF) [file pcbi.1007405.s004.tif]

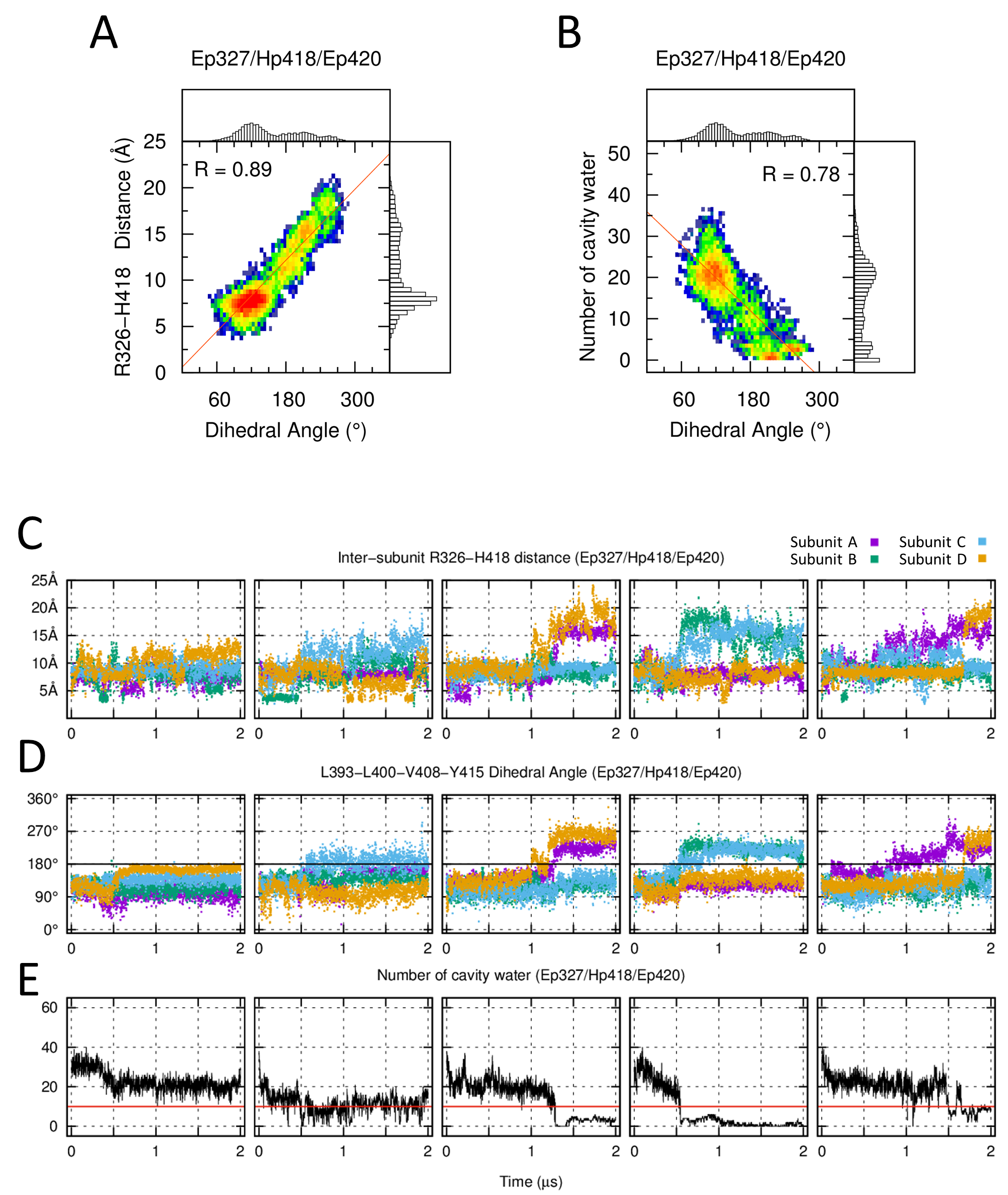

Supplement: S5 Fig — (A) Ensembles population of log for the R326–H418 distances and dihedral angles, and (B) ensembles population of log scale for the dihedral angles and number of water molecules in the water-filled cavity. The time evolution of (C) the distance between R326 and H418 residues, (D) the dihedral angle, and (E) the number of water molecules in the water cavity from five individual trajectories for Ep327/Hp418/Ep420. (TIF) [file pcbi.1007405.s005.tif]

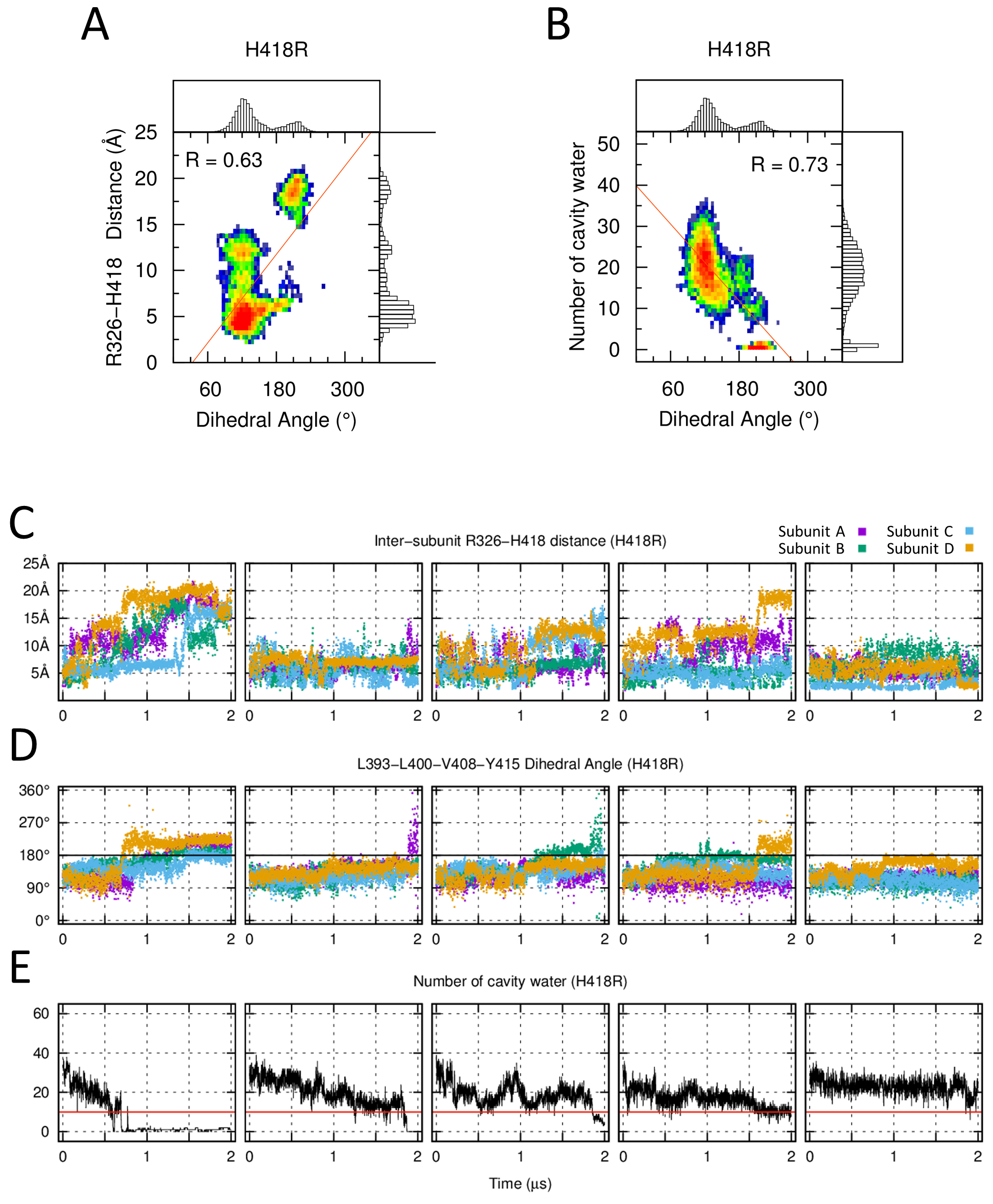

Supplement: S6 Fig — (A) Ensembles population of log for the R326–H418 distances and dihedral angles, and (B) ensembles population of log scale for the dihedral angles and number of water molecules in the water-filled cavity. The time evolution of (C) the distance between R326 and H418 residues, (D) the dihedral angle, and (E) the number of water molecules in the water cavity from five individual trajectories for H418R. (TIF) [file pcbi.1007405.s006.tif]

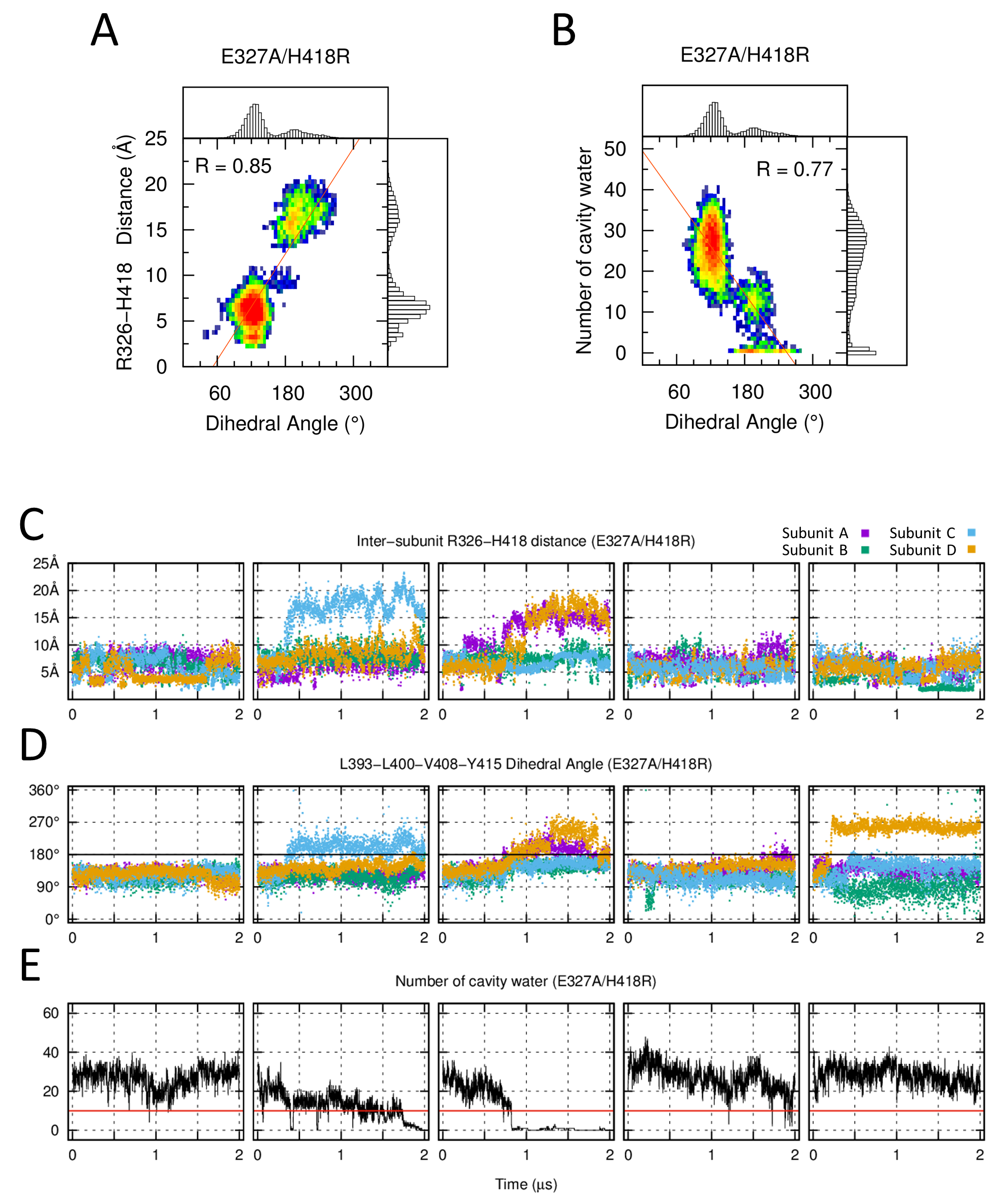

Supplement: S7 Fig — (A) Ensembles population of log for the R326–H418 distances and dihedral angles, and (B) ensembles population of log scale for the dihedral angles and number of water molecules in the water-filled cavity. The time evolution of (C) the distance between R326 and H418 residues, (D) the dihedral angle, and (E) the number of water molecules in the water cavity from five individual trajectories for E327A/H418R. (TIF) [file pcbi.1007405.s007.tif]

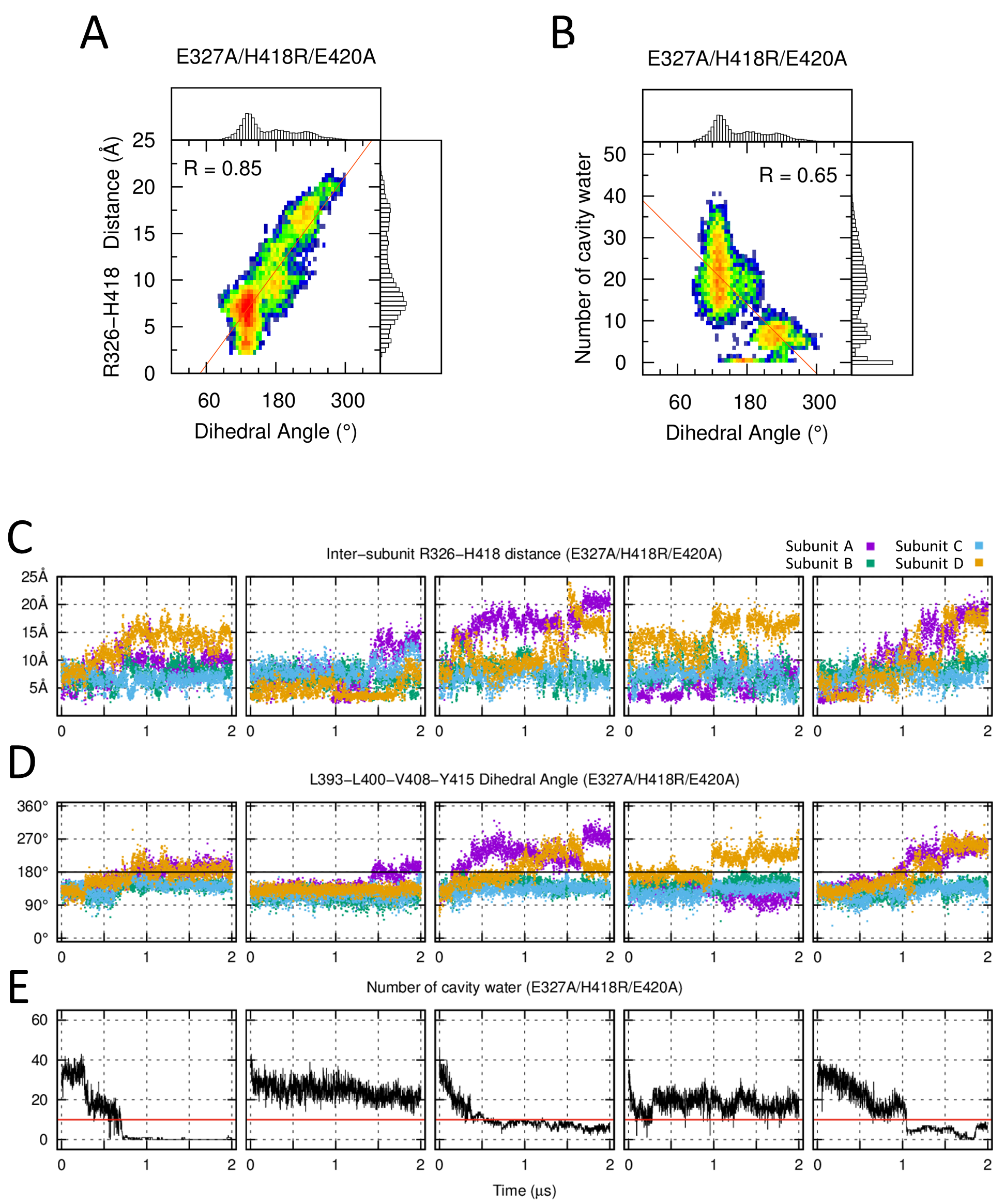

Supplement: S8 Fig — (A) Ensembles population of log for the R326–H418 distances and dihedral angles, and (B) ensembles population of log scale for the dihedral angles and number of water molecules in the water-filled cavity. The time evolution of (C) the distance between R326 and H418 residues, (D) the dihedral angle, and (E) the number of water molecules in the water cavity from five individual trajectories for E420A. (TIF) [file pcbi.1007405.s008.tif]

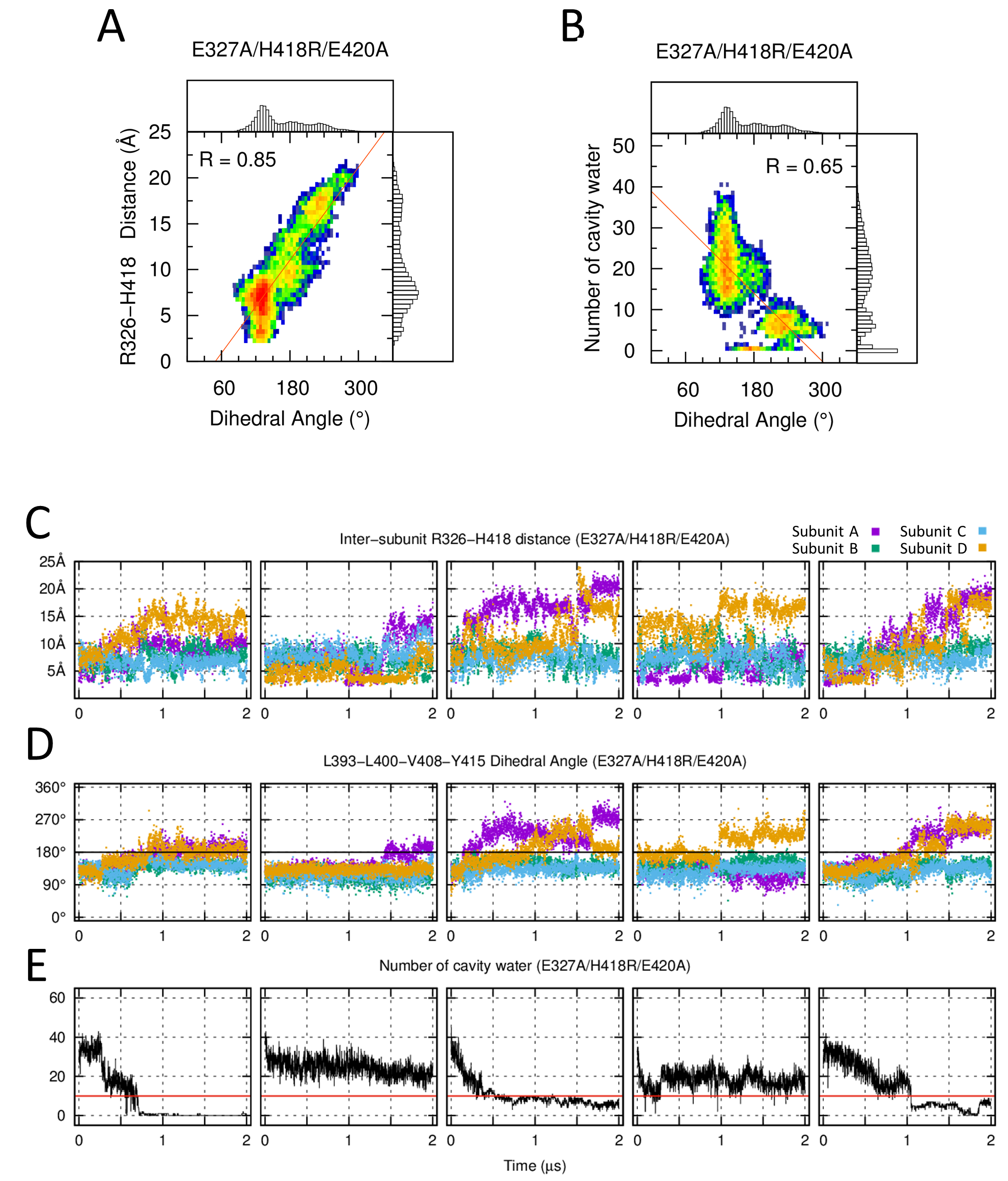

Supplement: S9 Fig — (A) Ensembles population of log for the R326–H418 distances and dihedral angles, and (B) ensembles population of log scale for the dihedral angles and number of water molecules in the water-filled cavity. The time evolution of (C) the distance between R326 and H418 residues, (D) the dihedral angle, and (E) the number of water molecules in the water cavity from five individual trajectories for E327A/H418R/E420A. (TIF) [file pcbi.1007405.s009.tif]
