# Supplementary material for: Structural and molecular insight into the pH-induced low-permeability of the voltage-gated potassium channel Kv1.2 through dewetting of the water cavity
Source: PLoS Comput Biol. 2020 Apr 21;16(4):e1007405. doi: 10.1371/journal.pcbi.1007405 (PMC7173763; doi:10.1371/journal.pcbi.1007405)
Supplement: Attachment — Submitted filename: Highlighted_final_Revision_20191224.docx [file pcbi.1007405.s010.docx]

•We provided all input files to run the MD simulations and all the trajectory files with google drive. But, the google drive was blocked so we provide the files with a different path.

We modified the README_Deposit files.pdf

**Before**

**Deposit files.**

**We performed MD simulations using GROMACS 5.0.6 that can be obtained from http://www.gromacs.org/Downloads_of_outdated_releases**

**We provide all input files to run the MD simulations including input coordinates, topologies and parameter files and etc. Additionally, we provide all the trajectory files.**

**Input files:**

https://drive.google.com/drive/folders/1QkgxMESPfS1wnd7mDdF2WtOnElE5Yy3W?usp=sharing

**Trajectory files**

**00_Wild-type**

**Trajectory 1:** https://drive.google.com/file/d/1VBpTaV9gqsEO9pqsc9bkDC_l8iDnM4eF/view?usp=sharing

…

==============================================================================

**After**

**Deposit files**

We performed MD simulations using GROMACS 5.0.6 that can be obtained from http://www.gromacs.org/Downloads_of_outdated_releases

We provide all input files to run the MD simulations including input coordinates, topologies and parameter files and etc. Additionally, we provide all the trajectory files.

**Input files:** http://wyu.dgist.ac.kr/kv12/input/

**Trajectory files:** http://wyu.dgist.ac.kr/kv12/traj/

**Reviewer #1:**

The authors investigate the pH-dependent gating mechanism for the pore domain of Kv1.2 channel. The authors report that a decrease in pH, leads to protonation of residues H418 and E327. The resulting repulsive coulombic interactions between R326 and Hp418 straightens the S6 helices, leading to de-wetting of the internal cavity and channel closure. These findings could help to better understand the role of pH in the gating mechanism of channels from the Kv family in health and disease conditions.
I would like the authors to address the following comments:

***The authors refer to the deprotonated and protonated states as wild-type and Ep327/Hp418, respectively. This naming should be corrected. Both, the deprotonated and protonated states are “wild-type” if no aminoacid-residue substitution was performed.**

- We change the name from Wild-type to Wild^UnP^, which means “Unprotonated wild type”.

**Before the change (in results part).**

• Because only the open conformations of the Kv1.2 channel were available from the Protein Data Bank [14]

•We performed atomistic MD simulations of the central pore domain in the Kv1.2 channel for both a Ep327/Hp418 state and a wild-type (as a control group) (here, “p” indicates the protonation; details of our MD simulations are provided in the Supplementary Information).

…

==============================================================================

**After the change (in results part).**

• Nevertheless, the unprotonated wild-type (named Wild^UnP^) conformation provided by PDB 2R9R [14] was in the open conformation.

• We performed atomistic MD simulations of the central pore domain in the Kv1.2 channel for both a Ep327/Hp418 state (here, “p” indicates the protonation; details of our MD simulations are provided in the Supplementary Information) and Wild^UnP^ (as a control group).

…

***On page 15, the authors mention E327/H418R variant, but according to table 1, it should be E327A/H418R double-mutant.**

- We corrected the typo error.

**Before the change (in results part).**

•E327/H418R mutant mimics the effect of acidic pH conditions. As expected, our simulation results showed that the pore domain of the mutant prefers the closed conformations compared with the wild-type Kv1.2 channel (Table 1).

==============================================================================

**After the change (in results part).**

•E327A/H418R mutant mimics the effect of acidic pH conditions. As expected, our simulation results showed that the closed conformations were more frequently sampled in E327A/H418R mutant (1807 snapshots) compared to those of the Wild^UnP^ (0 snapshots) in Table 1.

***The authors should make clear their definition of permeability. From the paper, I understand that it is based on the access of water molecules to the internal cavity of the channel. However, it can be confused with ion permeation events, for which the authors didn’t report any occurrence.**

- We modified the sentence in the introduction part as following

**Before the change (in introduction part).**

•The ionic currents are intimately connected to the permeability of the ions in the channels.

==============================================================================

**After the change (in introduction part).**

•A permeability of ion channels is defined by the ionic currents per surface area. The appearance of ionic currents is induced by the opening transformation of the channels with wetting water cavity. On the other hand, the disappearance of ionic currents is accompanied by the closing transformation of the channels with dewetting cavity [7].

***The authors claim to have modified “genetically” the permeability of Kv1.2 via mutation of key residues (page 16). This can be understood as if the authors performed experimental mutagenesis, which they didn’t. Hence, I would recommend using “in silico” instead of “genetically” in this sentence to avoid confusion.**

- Thank you for your comment, we modified as you mentioned.

**Before the change (in results part).**

•In our simulation, H418R and E327A/H418R mutants have low ion permeability, even under the neutral pH condition, and we were able to genetically modulate the ion permeability of Kv1.2 via mutation of the key residues.

==============================================================================

**After the change (in results part).**

•Thus, we were able to modulate the conformation of Kv1.2 via in silico mutation of the key residues. It imply that H418R and E327A/H418R mutants have low ion permeability, even under the neutral pH condition.

***The authors should discuss if pH changes not only affect gating by leading to closure of the pore domain as they propose. For instance should pH-decrease also affect voltage sensing and gating through modification of protonation states of key residues at the voltage-sensor domain of this channel?
Regarding methodologies:**

- The previous study shows that the S4 helix is the main moving part in the voltage-sensing domain (VSD) which moved down ~15Å across the membrane during the deactivation, and the motion of S4 affects the pore domain through the S4-S5 linker (Figure A) *(ref. : Jensen MØ, Jogini V, Borhani DW, Leffler AE, Dror RO, Shaw DE. Mechanism of voltage gating in potassium channels. Science. 2012;336(6078):229-33.)*

We discuss that VSD and pore domain communicate through the S4-S5 linker which is affected by the acidic pH condition. As a result, S4-S5 linker is affected by the applied voltage as well as the environmental pH.

**Before the change (in discussion part).**

•The molecular mechanism of the voltage-dependent gating of the Kv channels involves displacement of the voltage-sensing domains that regulate the wetting or dewetting of the cavity [7]. Therefore, the coexistence of these voltage- and acid-dependent gating mechanisms in Kv1.2 channels implies that both the voltage-induced structural pressure and acid-induced structural pressure can simultaneously influence the gating of the channels.

==============================================================================

**After the change (in discussion part).**

• The molecular mechanism of the voltage-dependent gating of the Kv channels involves displacement of the VSD that regulate the wetting or dewetting of the cavity [7]. The previous study shows that the S4 helix is the main moving part in the VSD which moved down ~15Å across the membrane during the deactivation, and the motion of S4 affects the pore domain through the S4-S5 linker [7]. In our simulation, the intra-subunit interaction between K312 and E420 is affected by the protonation state of E420, so the K312 (locate at the S4-S5 linker) could restrict the motion of S4 depending on the environmental pH condition. As a result, S4-S5 linker is affected by environmental pH. Since we simulated with the pore domain (residues 312–421, without VSDs) of Kv1.2, it does not fully reflect the realistic motion of a full chain of Kv1.2 with VSDs. However, it would be used to understand the effect of the acidic environment on channel gating of the pore domain. The coexistence of these voltage- and acid-dependent gating mechanisms in Kv1.2 channels implies that both the voltage-induced structural pressure and acid-induced structural pressure can simultaneously influence the gating of the channels.

**Figure A.** The motion of VSD during the gating. (Figure 1B and Figure 3 in *ref. Jensen MØ, Jogini V, Borhani DW, Leffler AE, Dror RO, Shaw DE. Mechanism of voltage gating in potassium channels. Science. 2012;336(6078):229-33.)*

***It is not clear why the authors used the Ep327/Hp418/Ep420 MD simulation for pKa estimates. Why not to use the MD simulation of the wild-type instead?**

-The reason of using the pore closure ensembles for pKa estimates was because the channel is in the closed state in the physiological condition at the resting voltage. We regarded the resting voltage as a default voltage and also the closed state as a default state of the channel. Since only the opened structure of Kv1.2 channel was available in Protein Data Bank (PDB: 2R9R) prior to our MD simulation, we needed to generate the closed structure of Kv1.2 channel from the opened structure PDB: 2R9R either by applying the resting voltage (about -60mV) or the acidic pH to an initial opened PDB: 2R9R structure. We, however, noted that our MD simulation was done only with the pore domain of the channel while the voltage sensing domain (VSD) of the channel is necessary to induce such conformational change subject to the application of the resting voltage. In our setting of MD simulation, we therefore applied the strong acidic condition (namely protonating E327/H418/E420) to an initial opened PDB: 2R9R structure in order to obtain the closed state in the physiological condition for Kv1.2 pore domain. Having done this process, we estimated the pKa values of titratable residues in Kv1.2 channel with the pore closure ensembles.

The reference (*Ishii K, Nunoki K, Yamagishi T, Okada H, Taira N. Differential sensitivity of Kv1. 4, Kv1. 2, and their tandem channel to acidic pH: involvement of a histidine residue in high sensitivity to acidic pH. Journal of Pharmacology and Experimental Therapeutics. 2001;296(2):405-11.*) states that Kv1.2 channels in the resting (depolarizing) voltage are inactivated (activated) with zero (finite) permeability which is independent (dependent) to pH conditions. The finite permeability is determined by the ratio between the number of open and close channels, and is reduced under acidic pH condition. Therefore, we tried to analyze both the open-to-close ratio and the molecular mechanism of the pore closure by protonated residues.

We modified the following sentence.

**Before the change (in results part).**

• To identify the key residues that might be protonated under acidic conditions, that is, from pH 7.5 to around pH 5.3, we attempted to estimate the pKa values of the titratable residues in Kv1.2 channels with closed conformations. Because only the open conformations of the Kv1.2 channel were available from the Protein Data Bank [14],

==============================================================================

**After the change (in results part).**

• To identify the key residues that might be protonated under acidic pH and resting voltage conditions, that is, from pH 7.5 to around pH 5.3, we attempted to estimate the pKa values of the titratable residues in Kv1.2 channels with closed conformations. The Kv1.2 channel is inactivated with zero permeability in the resting voltage [12]. Thus, physiologically the default state of the Kv1.2 channel is an inactivated state with the pore closure. Nevertheless, the Wild^UnP^ conformation provided by PDB 2R9R [14] was in the open conformation. Therefore, we needed to generate the closed structure of Kv1.2 channel from the opened structure either by applying the resting voltage (about -60mV) or the acidic pH to an initial opened structure. We, however, noted that our MD simulation was done only with the pore domain of the channel while the voltage sensing domain (VSD) of the channel is necessary to induce such conformational change subject to the application of the resting voltage. In our setting of MD simulation, we therefore applied the strong acidic condition (namely protonating E327/H418/E420) to an initial opened structure in order to obtain the closed state in the physiological condition for Kv1.2 pore domain.

***Could the authors discuss why the pKa analysis shows that all four H418 residues could be protonated but only two of the four E327 residues?**

- As shown in the upper panels of Fig 3A, the closed state of the Kv1.2 channel has the 2-fold symmetry instead of 4-fold symmetry with the open state. Thus, we think that four E327 residues sensitively react to the structural change to 2-fold symmetry rather than the other titratable residues.

We inserted the described part for the loss of 4-fold symmetry as following

**Before the change (in results part).**

•The pKa values of both E327 in the A- and D-chain and H418 were around 6.0, which means that these two residues can be protonated under the above acidic conditions.

==============================================================================

**After the change (in results part).**

•The pKa values of both E327 in the A- and D-chain and H418 were around 6.0, which means that these two residues can be protonated under the above acidic conditions. Here, pKa of E327 residues were seemed to reside in the 2-fold symmetry whereas pKa of H418 residues remained in the 4-fold symmetry. The closed conformations of the channel, considered for our pKa estimations, were shown to maintain 2-fold symmetry near the water cavity, while the other parts of the channel were remained in 4-fold symmetry (Upper panels of Fig 3A). Presumably, the pKa of E327 residues sensitively reacted to the structural change near the water cavity with 2-fold symmetry rather the pKa of H418 residues.

***The statistical significance of the results is not clear. For instance, is 3/5 closing events statistically different from 2/5, 1/5 or 0/5 closing events? The authors should increase the number of replicas per system and use some statistical figure or metric for comparison.**

- It is meaningful to check the statistical significance of the results by increasing the number of replicas. But we want to focus on explaining the structural changes and phenomena that can occur in an acidic environment in the pore domain of Kv1.2. Additional difficulty in increasing the number of replicas is that the MD simulation speed is 30ns/day with 20 CPU and 1GPU for each replica. We ran the simulation with 45 replicas (9 system *5 replicas). For our 2μs simulations, 900 CPU and 45 GPU are needed for 67days.

So, we change the Table 1 in terms of the number of structural ensembles and sentence for closing events

| **Closed (N/2000)** | **#1** | **#2** | **#3** | **#4** | **#5** | **Sum** |
| --- | --- | --- | --- | --- | --- | --- |
| **Wild^UnP^** | 0 | 0 | 0 | 0 | 0 | 0 |
| **Hp418** | 0 | 7 | 342 | 938 | 0 | 1287 |
| **Ep327/Hp418** | 1056 | 987 | 871 | 1 | 0 | 2915 |
| **Ep420** | 0 | 60 | 0 | 687 | 0 | 747 |
| **Ep327/Hp418/Ep420** | 0 | 687 | 724 | 1458 | 395 | 3264 |
| **H418R** | 1350 | 202 | 157 | 124 | 2 | 1835 |
| **E327A/H418R** | 3 | 601 | 1186 | 17 | 0 | 1807 |
| **E420A** | 187 | 10 | 0 | 0 | 0 | 197 |
| **E327A/H418R/E420A** | 1342 | 0 | 1321 | 47 | 960 | 3670 |

**Table 1. The number of snapshots with less than 10 water in the cavity in all MD simulations.**

**Before the change (in results part).**

•The time evolution of the number of water molecules in the cavity of the pore showed that the conformations from three trajectories of the Ep327/Hp418 state equilibrated to the closed conformations with the dewetted cavity, whereas those from all five trajectories of the wild-type remained in the open conformations with the wetted cavity (Figs 3B and 3C) [16].

•E327/H418R mutant mimics the effect of acidic pH conditions. As expected, our simulation results showed that the pore domain of the mutant prefers the closed conformations compared with the wild-type Kv1.2 channel (Table 1).

•As expected, our simulated H418R mutant preferred the closed conformations compared with the wild-type Kv1.2 channel (Table 1).

==============================================================================

**After the change (in results part).**

•The number of water molecules in the cavity implicates that the closed conformations were more frequently sampled in Ep327/Hp418 (2915 snapshot) compared to those of Wild^UnP^ (0 snapshot) in Table 1 [17]. This result demonstrates that the protonation of E327 and H418 destabilizes the charge interaction network of R326, E327, and H418 under acidic condition. As a result, it induces conformational change from open to close form (Fig 3B and 3C).

•E327A/H418R mutant mimics the effect of acidic pH conditions. As expected, our simulation results showed that the closed conformations were more frequently sampled in E327A/H418R mutant (1807 snapshots) compared to those of the Wild^UnP^ (0 snapshots) in Table 1.

•As expected, our simulation results showed that the closed conformations were more frequently sampled in H418R mutant (1835 snapshot) compared to those of the Wild^UnP^ (0 snapshot) in Table 1.

***For each system, were the replicas run from different initial conditions?**

- For each system, the initial conditions are the same in all replicas except the initial velocities for each atom, which are assigned with a random number generator depending on the setting temperature (T=310K) by a standard protocol in MD simulation.

**Reviewer #2: Review of PCOMPBIOL-D-19-01546**

This is a potentially interesting computational study of pH-induced low-permeability of Kv1.2 channels. The authors suggest on the basis of simulations that acid-induced reduction in permeability of this channel is due to protonation of E327 and H148 which in turn leads to loss of curvature/bending of the helices and a consequent dewetting of water-filled cavity. This would be an interesting result. However I have some questions about the underlying logic of how the simulations were designed and performed.

**The pKa calculations use MEAD – does this work for membrane proteins? How is the bilayer modelled (e.g. as a region of low dielectric)? This needs to be made explicit. The description in the Methods (page 17) is very unclear – I am still uncertain as to what was actually done even after reading is several times. It also fails to mention that the use of red circles and blue crosses refers to Fig. 2A which does not help.**

- We modified the paragraph in the methods part as following

**Before the change (in methods part).**

•For pKa estimates of H418 and the acidic residues in the pore domain of the closed Kv1.2 channels, we extracted 98 ensemble structures in the last 1-μs time window from our MD simulation for a Ep327/Hp418/Ep420 state. In addition, after applying MEAD programs [15], we selected 765 titration states of the channels that were predominant in the pH range from 3 to 8. At each titration state, we calculated the energy for each of 98 ensemble structures, as well as their average and standard deviation using AMBER force field 99SB [31]. Red circles in the figure represent the pKa values when the average energies of ensemble structures, $\left\langle E \right\rangle$, were used to calculate the titration fraction as a function of pH for the corresponding titration states. titration states. ==============================================================================

**After the change (in methods part).**

•For pKa estimates of H418 and the acidic residues in the pore domain of the closed Kv1.2 channels, several processes were executed. 1) We extracted 98 ensemble structures of the Kv1.2 channels with pore closure in the last 1 μs time window from our MD simulation for an Ep327/Hp418/Ep420 state. In our setting of MD simulation the pore domain of Kv1.2 channel together with explicit membrane lipids, water molecules, and ions were included. 2) In order to select preferred titration states among all possible titration states in the pH range from 3 to 8, we took advantage of MEAD algorithms [15] to the 10 out of 98 pore closure ensembles with these options (dielectric constants of a molecular interior region / solvent region were $\varepsilon_{\text{in}}=6.0, \varepsilon_{\text{sol}}=80.0$, ionic strength was 0.15 mol/l, and the effect of membrane was excluded.). 3) We selected 765 titration states of the channels that were predominant in the pH range from 3 to 8. For the estimation of pKa values we utilized these 765 titration states and 10 pore closure structural ensembles from our MD simulation. 4) At each titration state on all 98 ensemble structures, we calculated the system energies in the implicit water using AMBER force field 99SB [31]. 5) From the titration states of 3) and the system energies of 4), we reconstructed the partition function, the protonated fractions and estimated pKa values of H418 and the acidic residues (Fig 2A) [33]. Red circles in the figure represent the pKa values when the average energies (using AMBER force field 99SB) of ensemble structures, $\left\langle E \right\rangle$, were used to calculate the protonation fraction as a function of pH for the corresponding titration states.

**From the main Results text (page 12) and Fig. 2 it is stated that “pKa values of both E327 in the A- and D-chain and H418 were around 6.0”. From Fig. 2A it can be seen that the values for E327 of the B & C chains are ~4.5. To me this implies there must have been loss of 4-fold symmetry in the closed state model – this should be described and discussed.**

- We inserted the described part for the loss of 4-fold symmetry as following

**Before the change (in results part).**

•The pKa values of both E327 in the A- and D-chain and H418 were around 6.0, which means that these two residues can be protonated under the above acidic conditions.

==============================================================================

**After the change (in results part).**

•The pKa values of both E327 in the A- and D-chain and H418 were around 6.0, which means that these two residues can be protonated under the above acidic conditions. Here, pKa of E327 residues were seemed to reside in the 2-fold symmetry whereas pKa of H418 residues remained in the 4-fold symmetry. The closed conformations of the channel, considered for our pKa estimations, were shown to maintain 2-fold symmetry near the water cavity, while the other parts of the channel were remained in 4-fold symmetry (Upper panels of Fig 3A). Presumably, the pKa of E327 residues sensitively reacted to the structural change near the water cavity with 2-fold symmetry rather the pKa of H418 residues.

**After describing the pKas for the closed state (model) they then go back to the open state structure (page 13), protonate E327 and H418 (earlier on they also protonated E420, page 12) and show that the channel closes. What is the relationship of the Ep327/Hp418 closed state model to the previous Ep327/Hp418/Ep420 model? Are they the same? Do they show comparable breakdown of 4-fold symmetry.**

- The closed state of Ep327/Hp418 with breakdown of 4-fold symmetry looks similar with that of Ep327/Hp418/Ep420. And we explain the logic of our pKa estimation and MD simulations in the next answer.

**This then leads me to question the logic of the design of the simulations. Would it not be more logical to start with the (experimental) open state, calculate pKas, decide on a protonation state for lowered pH and then run the simulations to see if the channel closes? This seems to me to be a major flaw in the design of the study, and needs to be addressed directly by running the open state pKa calculations and simulations based on these (or at least explaining the logic of the simulation design used).**

-The reason of using the pore closure ensembles for pKa estimates was because the channel is in the closed state in the physiological condition at the resting voltage. We regarded the resting voltage as a default voltage and also the closed state as a default state of the channel. Since only the opened structure of Kv1.2 channel was available in Protein Data Bank (PDB: 2R9R) prior to our MD simulation, we needed to generate the closed structure of Kv1.2 channel from the opened structure PDB: 2R9R either by applying the resting voltage (about -60mV) or the acidic pH to an initial opened PDB: 2R9R structure. We, however, noted that our MD simulation was done only with the pore domain of the channel while the voltage sensing domain (VSD) of the channel is necessary to induce such conformational change subject to the application of the resting voltage. In our setting of MD simulation, we therefore applied the strong acidic condition (namely protonating E327/H418/E420) to an initial opened PDB: 2R9R structure in order to obtain the closed state in the physiological condition for Kv1.2 pore domain. Having done this process, we estimated the pKa values of titratable residues in Kv1.2 channel with the pore closure ensembles.

The reference (*Ishii K, Nunoki K, Yamagishi T, Okada H, Taira N. Differential sensitivity of Kv1. 4, Kv1. 2, and their tandem channel to acidic pH: involvement of a histidine residue in high sensitivity to acidic pH. Journal of Pharmacology and Experimental Therapeutics. 2001;296(2):405-11.*) states that Kv1.2 channels in the resting (depolarizing) voltage are inactivated (activated) with zero (finite) permeability which is independent (dependent) to pH conditions. The finite permeability is determined by the ratio between the number of open and close channels, and is reduced under acidic pH condition. Therefore, we tried to analyze both the open-to-close ratio and the molecular mechanism of the pore closure by protonated residues.

We modified the following sentence.

**Before the change (in results part).**

•To identify the key residues that might be protonated under acidic conditions, that is, from pH 7.5 to around pH 5.3, we attempted to estimate the pKa values of the titratable residues in Kv1.2 channels with closed conformations. Because only the open conformations of the Kv1.2 channel were available from the Protein Data Bank [14],

==============================================================================

**After the change (in results part).**

•To identify the key residues that might be protonated under acidic pH and resting voltage conditions, that is, from pH 7.5 to around pH 5.3, we attempted to estimate the pKa values of the titratable residues in Kv1.2 channels with closed conformations. The Kv1.2 channel is inactivated with zero permeability in the resting voltage [12]. Thus, physiologically the default state of the Kv1.2 channel is an inactivated state with the pore closure. Nevertheless, the Wild^UnP^ conformation provided by PDB 2R9R [14] was in the open conformation. Therefore, we needed to generate the closed structure of Kv1.2 channel from the opened structure either by applying the resting voltage (about -60mV) or the acidic pH to an initial opened structure. We, however, noted that our MD simulation was done only with the pore domain of the channel while the voltage sensing domain (VSD) of the channel is necessary to induce such conformational change subject to the application of the resting voltage. In our setting of MD simulation, we therefore applied the strong acidic condition (namely protonating E327/H418/E420) to an initial opened structure in order to obtain the closed state in the physiological condition for Kv1.2 pore domain.

I have a couple of more technical points:

**Fig. 3 – This describes pore closure. It would be helpful to have pore radius profiles shown for that start and end of each simulation. These should reveal to what extent dewetting is the consequence of pore narrowing/occlusion.**

- Thank you for your comment, we measured the pore radius and modified Figure 3A.

**Fig. 4 – I suggest better descriptions of the S6 helix would be ‘bent’ and ‘straight’. However, it looks to me that even after ‘straightening’ the S6 helix remains kinked, but in a different direction. Helix kink angles can be quantified and perhaps this is needed to assess the reproducibility of the open -> closed transition in the various simulations.**

- Thank you for your comment, we modified Figure 4A.

We measured the kink angle via “the dihedral angle” which is extended by the positions of the Cα atoms in four residues (L393, L400, V408, and Y415) on the S6 helix (yellow ball of the side view in Fig 4A). We measured dihedral angle over time for all simulation (Fig S1E~S9E)

**Before the change (in results part).**

•the dihedral angle extended by the positions of the Cα atoms in four residues (L393, L400, V408, and Y415) on the S6 helix (the side view in Fig 4A). The dihedral angle determines whether the S6 helix is bent or straight [19].

==============================================================================

**After the change (in results part).**

•the dihedral angle extended by the positions of the Cα atoms in four residues (L393, L400, V408, and Y415) on the S6 helix (yellow ball of the side view in Fig 4A) as a measure of the kinked angle of S6. The dihedral angle determines whether the S6 helix is bent or straight [18].

**Reviewer #3:**

This manuscript presents a molecular dynamics study of voltage-gated K+ channel Kv1.2. The authors investigate the effect of protonating residues located on the intracellular side of the channel on channel closure. Protonating two residues, E327 and H418, results in the loss of electrostatic interactions at the end of the S6 helix and correlate with S6 straightening and pore dewetting, providing molecular insight into the observed reduction in ion current at low pH.

The paper is interesting and the work appears to be well executed, but clarifying the rationale for the calculations and extending the analysis of the results presented to all the systems studied would strengthen the paper. In particular:

**1. The rationale for the simulations is unclear. Why was only the closed state of the channel considered for pKa calculations? If conduction is lower but not abolished at low pH, doesn't that suggest that the channel still has a significant propensity to be open?**

The reason of using the pore closure ensembles for pKa estimates was because the channel is in the closed state in the physiological condition at the resting voltage. We regarded the resting voltage as a default voltage and also the closed state as a default state of the channel. Since only the opened structure of Kv1.2 channel was available in Protein Data Bank (PDB: 2R9R) prior to our MD simulation, we needed to generate the closed structure of Kv1.2 channel from the opened structure PDB: 2R9R either by applying the resting voltage (about -60mV) or the acidic pH to an initial opened PDB: 2R9R structure. We, however, noted that our MD simulation was done only with the pore domain of the channel while the voltage sensing domain (VSD) of the channel is necessary to induce such conformational change subject to the application of the resting voltage. In our setting of MD simulation, we therefore applied the strong acidic condition (namely protonating E327/H418/E420) to an initial opened PDB: 2R9R structure in order to obtain the closed state in the physiological condition for Kv1.2 pore domain. Having done this process, we estimated the pKa values of titratable residues in Kv1.2 channel with the pore closure ensembles.

The reference (*Ishii K, Nunoki K, Yamagishi T, Okada H, Taira N. Differential sensitivity of Kv1. 4, Kv1. 2, and their tandem channel to acidic pH: involvement of a histidine residue in high sensitivity to acidic pH. Journal of Pharmacology and Experimental Therapeutics. 2001;296(2):405-11.*) states that Kv1.2 channels in the resting (depolarizing) voltage are inactivated (activated) with zero (finite) permeability which is independent (dependent) to pH conditions. The finite permeability is determined by the ratio between the number of open and close channels, and is reduced under acidic pH condition. Therefore, we tried to analyze both the open-to-close ratio and the molecular mechanism of the pore closure by protonated residues.

We modified the following sentence.

**Before the change (in results part).**

•To identify the key residues that might be protonated under acidic conditions, that is, from pH 7.5 to around pH 5.3, we attempted to estimate the pKa values of the titratable residues in Kv1.2 channels with closed conformations. Because only the open conformations of the Kv1.2 channel were available from the Protein Data Bank [14],

==============================================================================

**After the change (in results part).**

•To identify the key residues that might be protonated under acidic pH and resting voltage conditions, that is, from pH 7.5 to around pH 5.3, we attempted to estimate the pKa values of the titratable residues in Kv1.2 channels with closed conformations. The Kv1.2 channel is inactivated with zero permeability in the resting voltage [12]. Thus, physiologically the default state of the Kv1.2 channel is an inactivated state with the pore closure. Nevertheless, the Wild^UnP^ conformation provided by PDB 2R9R [14] was in the open conformation. Therefore, we needed to generate the closed structure of Kv1.2 channel from the opened structure either by applying the resting voltage (about -60mV) or the acidic pH to an initial opened structure. We, however, noted that our MD simulation was done only with the pore domain of the channel while the voltage sensing domain (VSD) of the channel is necessary to induce such conformational change subject to the application of the resting voltage. In our setting of MD simulation, we therefore applied the strong acidic condition (namely protonating E327/H418/E420) to an initial opened structure in order to obtain the closed state in the physiological condition for Kv1.2 pore domain.

**2. Why were only two out of four copies of E327 estimated to have a higher pKa? On the one hand, this result indicates that the simulations have not converged (the four residues should have identical pKa). On the other hand, if taken at face value, this result questions the rationale for protonating all four E327 residues in the subsequent simulations.**

- As shown in the upper panels of Fig 3A, the closed state of the Kv1.2 channel has the 2-fold symmetry instead of 4-fold symmetry with the open state. Thus, we think that four E327 residues sensitively react to the structural change to 2-fold symmetry rather than the other titratable residues. Additionally, previous simulation studies vividly showed that the 2-fold symmetric closed stricture is a stable structure. (See the blue color in Figure 2B) *(ref. : Jensen MØ, Jogini V, Borhani DW, Leffler AE, Dror RO, Shaw DE. Mechanism of voltage gating in potassium channels. Science. 2012;336(6078):229-33, Jensen MO, Borhani DW, Lindorff-Larsen K, Maragakis P, Jogini V, Eastwood MP, et al. Principles of conduction and hydrophobic gating in K+ channels. Proc Natl Acad Sci U S A. 2010;107(13):5833-8.)*

One may wonder the pKa of E327 shows 2-fold symmetry, but the pore opened initial structure for MD simulation shows 4-fold symmetry. In fact, all of protonation states in our MD simulations (Hp418, Ep327/Hp418, and Ep420 or Ep327/Hp418/Ep420) are with 4-fold symmetry. Thus, our results didn’t draw a picture of the channel under specific pH. Nevertheless, it is important that we observed the increasing propensity in the number of closed ensembles of the protonated states, Hp418 or Ep327/Hp418 or Ep420 or Ep327/Hp418/Ep420, rather than unprotonated state.

We inserted the described part for the loss of 4-fold symmetry as following

**Before the change (in results part).**

•The pKa values of both E327 in the A- and D-chain and H418 were around 6.0, which means that these two residues can be protonated under the above acidic conditions.

==============================================================================

**After the change (in results part).**

•The pKa values of both E327 in the A- and D-chain and H418 were around 6.0, which means that these two residues can be protonated under the above acidic conditions. Here, pKa of E327 residues were seemed to reside in the 2-fold symmetry whereas pKa of H418 residues remained in the 4-fold symmetry. The closed conformations of the channel, considered for our pKa estimations, were shown to maintain 2-fold symmetry near the water cavity, while the other parts of the channel were remained in 4-fold symmetry (Upper panels of Fig 3A). Presumably, the pKa of E327 residues sensitively reacted to the structural change near the water cavity with 2-fold symmetry rather the pKa of H418 residues.

**Figure B.** Channel closure, intracellular view; only the protein is shown. VSD helices S1-S3a, green; VSD helices S3b-S4 ("paddle"), red; VSD-pore (S4-S5) linker helix, orange; pore domain, white; Pro405-Val406-Pro407 ("PVP motif") pore constriction, blue. Applied voltage is -500mV. (Movie S1 in *ref. Jensen MØ, Jogini V, Borhani DW, Leffler AE, Dror RO, Shaw DE. Mechanism of voltage gating in potassium channels. Science. 2012;336(6078):229-33.)*

**3. Most importantly, the manuscript indicates that as many as 8 variants of the channel were simulated (Table 1), but detailed analysis is only reported for 4 of them (Ep/Hp, EA/HR, HR, and Ep/Hp/Ep), and, surprisingly, a full analysis of the results is only provided for the Ep/Hp system without any justification for neglecting the other systems. In particular, full analysis of the above as well as the other channel variants in which dewetting was observed (namely, Ep, Hp, EA and EA/HR/EA) should be provided in order to test the validity and the generality of the mechanism proposed by the authors, linking the changes in electrostatic interactions to helix straightening and pore dewetting.
In other words, limiting the full analysis to doubly-protonated E327 and H418 is not justified. Since all the other variants lead to pore dewetting, they should all be analyzed to the same extent as the wild type and Ep/Hp variants.**

-Thank you for your comment, we provide the details of the full analysis for all variants and show the results in Fig S1~S9.

**Before the change (in results part).**

•The permeability of the Kv1.2 channel was further probed by examining the changes in the inter-subunit interactions among R326, E327, and H418 and the intra-subunit interaction between K312 and E420. We were able to modify the charge states of these residues through protonation or mutation, as shown in Table 1. First, we modified the inter-subunit interaction by changing the charge state of H418 to resemble the weak acidic condition. Second, we changed the charge states of both E327 and H418 to break the inter-subunit interaction, which resembled the stronger acidic condition. Third, we modified the intra-subunit interaction by changing the charge state of E420. Finally, we broke both the inter- and intra-subunit interactions by simultaneously changing the charge state of E327, H418, and E420, which resembled the strong acidic condition. Our simulation results showed that the transition from the open to the closed conformation in the pore of the Kv1.2 channel depends on the state of the inter-subunit interaction (Table 1). The E327 and H418 residues located on the intracellular side across the membrane have a major effect on the pH-sensing mechanism, depending on their protonation states.

==============================================================================

**After the change (in results part).**

•The conformation of the Kv1.2 channel was further probed by examining the changes in the inter-subunit interactions among R326, E327, and H418 and the intra-subunit interaction between K312 and E420. We were able to modify the charge states of these residues through protonation or mutation, as shown in Table 1. First, we modified the inter-subunit interaction by changing the charge state of H418 to resemble the weak acidic condition (Fig S2 and S6). Second, we changed the charge states of both E327 and H418 to break the inter-subunit interaction, which resembled the stronger acidic condition (Fig S3 and S7). Third, we modified the intra-subunit interaction by changing the charge state of E420 (Fig S4 and S8). Finally, we broke both the inter- and intra-subunit interactions by simultaneously changing the charge state of E327, H418, and E420, which resembled the strong acidic condition (Fig S5 and S9). Our simulation results showed that the transition from the open to the closed conformation in the pore of the Kv1.2 channel depends on the state of the inter-subunit interaction (Table 1). The E327 and H418 residues located on the intracellular side across the membrane have a major effect on the pH-sensing mechanism, depending on their protonation states.

**4. Contrary to the claim made by the authors, the multiple sequence alignment presented in Fig. 2B does not provide rigorous evidence that the 2 residues considered are highly conserved, since most residues are conserved in the sequences shown.**

-Thank you for your comment. We modified the following sentence about the multiple sequence alignment.

**Before the change (in results part).**

•Multiple sequence alignment for Kv1 subfamily proteins showed that these two residues are conserved (Fig 2B) and are located near the water-filled cavity (Fig 2C). Therefore, we decided to investigate the effect of protonation of the E327 and H418 residues on the conformational transition of the pore domain of the Kv1.2 channel.

==============================================================================

**After the change (in results part).**

•Multiple sequence alignment for Kv1 subfamily proteins showed that almost residues are conserved. Among these conserved residues two residues E327 and H418 are located near the water-filled cavity, and many charged amino acids are distributed around E327 and H418. Since the protonation of E327 and H418 residues could affect the conformational transition of the pore domain of the Kv1.2 channel, we decided to investigate the effect of protonation of the E327 and H418.

**5. It should be noted that the simulations have not reached equilibrium, since some trajectories led to dewetting while some others remained wetted. As such, the simulations do not provide a rigorous estimate of the relative stability of wetted and dewetted states. Although the fact that dewetting is observed in the doubly-protonated system and not in the wild type suggests that dewetting is favoured by double protonation, strictly speaking it does not rule out that dewetting could occur in the wild type over longer time scales—in other words, the apparent relative kinetic stability (or lack thereof) of the wetted state does not imply its thermodynamic stability (or lack thereof). Therefore, statements such as “This result demonstrates that the pore domain of Kv1.2 channels prefers the closed conformations under acidic conditions”, as well as multiple other statements regarding such “preference”, are not supported by the results provided. The text should be carefully revised to omit any implication that the simulations have reached equilibrium.**

-Thank you for your comment. We modified the following sentences.

**Before the change (in introduction part).**

•We suggest the molecular and structural mechanism underlying the acid-induced low permeability of Kv1.2 channels by uncovering the mechanism through which the channels, under acidic conditions, prefer the closed-pore state with the dewetted cavity than the open-pore state with the wetted cavity.

**Before the change (in results part).**

•five trajectories of MD simulations for each of a wild-type and a Ep327/Hp418 state were run for 2 μs, and the equilibrium configurations of the central pore domain in the Kv1.2 channel were obtained (Fig 3A). The time evolution of the number of water molecules in the cavity of the pore showed that the conformations from three trajectories of the Ep327/Hp418 state equilibrated to the closed conformations with the dewetted cavity, whereas those from all five trajectories of the wild-type remained in the open conformations with the wetted cavity (Figs 3B and 3C) [16]. This result demonstrates that the pore domain of Kv1.2 channels prefers the closed conformations under acidic conditions, whereas the open conformations are abundant under the neutral pH condition. Therefore, acidic conditions hamper the opening of the channel even under a depolarizing voltage and lower the ion permeability of the channel.

•E327A/H418R mutant mimics the effect of acidic pH conditions. As expected, our simulation results showed that the pore domain of the mutant prefers the closed conformations compared with the wild-type Kv1.2 channel (Table 1).

•As expected, our simulated H418R mutant preferred the closed conformations compared with the wild-type Kv1.2 channel (Table 1).

==============================================================================

**After the change (in introduction part).**

•We suggest the molecular and structural mechanism underlying the acid-induced low permeability of Kv1.2 channels by uncovering the mechanism under acidic conditions.

**After the change (in results part).**

• five trajectories of MD simulations for each of a Wild^UnP^ and a Ep327/Hp418 state were run for 2 μs, and the conformational ensembles of the central pore domain in the Kv1.2 channel were sampled (Fig 3A) [16]. The number of water molecules in the cavity showed that the closed conformations were more frequently sampled in Ep327/Hp418 (2915 snapshot) compared to those of Wild^UnP^ (0 snapshot) in Table 1 [17]. This result demonstrates that the protonation of E327 and H418 destabilizes the charge interaction network of R326, E327, and H418 under acidic condition. As a result, it induces conformational change from open to close form (Fig 3B and 3C).

•E327A/H418R mutant mimics the effect of acidic pH conditions. As expected, our simulation results showed that the closed conformations were more frequently sampled in E327A/H418R mutant (1807 snapshots) compared to those of the Wild^UnP^ (0 snapshots) in Table 1.

•As expected, our simulation results showed that the closed conformations were more frequently sampled in H418R mutant (1835 snapshot) compared to those of the Wild^UnP^ (0 snapshot) in Table 1.

**6. The text states: “The hinge of the S6 helix maintains electrostatic balance through two inter-subunit interactions of R326-H418 and E327-H418. These interactions stabilize the open conformation of the Kv1.2 pore domain under a neutral pH condition.” The “hinge” of S6 is not define. Moreover, the authors do not provide evidence that these are the only interactions that change upon closing of the channel. Finally, as noted in comment #3 above, the authors should provide corroborating evidence for this mechanism by analyzing the other systems that they simulated as positive controls.
In addition, note that in the interpretation of the results, correlation does not imply causality.**

- We define the hinge with PVP (P405-V406-P407) motif which located in the middle of H6. Additionally, we show that inter-subunit interaction (R326, E327, and H418) is more critical factor for channel gating than inter-subunit interaction (K312 and E420) with the simulations results.

We modified the following sentence.

**Before the change (in results part).**

•Based on the structural ensembles collected from our MD simulations, we quantified the structural alterations between the Wild^UnP^ and Ep327/Hp418 type of Kv1.2 channels. The hinge of the S6 helix maintains electrostatic balance through two inter-subunit interactions of R326–H418 and E327–H418. These interactions stabilize the open conformation of the Kv1.2 pore domain under a neutral pH condition (left panel of Fig 4A). We used two structural determinants that can distinguish the open conformation from the closed conformation of the pore in Kv1.2 channels, namely, the R326–H418 inter-subunit distance defined by the nearest inter-atom distances in these two residues (the bottom view in Fig 4A) and the dihedral angle extended by the positions of the Cα atoms in four residues (L393, L400, V408, and Y415) on the S6 helix (yellow ball of the side view in Fig 4A) as a measure of the kinked angle of S6. The dihedral angle determines whether the S6 helix is bent or straight [19].

==============================================================================

**After the change (in results part).**

• Based on the structural ensembles collected from our MD simulations, we quantified the structural alterations between the open and close form of Kv1.2 channels. The PVP (P405-V406-P407) motif which is located in the middle of H6 (Fig 1B) is flexible and acts as a hinge [17, 18]. This flexible hinge allows the change of kinked angle during channel gating. We compared how inter-subunit interaction (R326, E327, and H418) and intra-subunit interactions (K312 and E420) affect the channel gating (Fig 4A). As a result, the closed structures were more frequently observed when changing the inter-subunit interaction (Hp418: 1287 snapshots in Table 1) compared to those of changing the intra-subunit interaction (Ep420: 747 snapshots in Table 1). We used two structural determinants that can distinguish the open conformation from the closed conformation of the pore in Kv1.2 channels, namely, the R326–H418 inter-subunit distance defined by the nearest inter-atom distances in these two residues (the bottom view in Fig 4A) and the dihedral angle extended by the positions of the Cα atoms in four residues (L393, L400, V408, and Y415) on the S6 helix (yellow ball of the side view in Fig 4A) as a measure of the kinked angle of S6. The dihedral angle determines whether the S6 helix is bent or straight [18].

**7. What do the authors mean by “Hp418 was electrostatically pushed by R326 at the hinge of the S6 helix”? The helical hinge seems to be far from the residues in question and is not defined (see comment #6 above).**

- Thank you for your comment. We modified the following sentence.

**Before the change (in results part).**

•Overall, Hp418 was electrostatically pushed by R326 at the hinge of the S6 helix under acidic conditions. This changed the shape of the S6 helix from bent to straight.

==============================================================================

**After the change (in results part).**

•Overall, Hp418 was electrostatically pushed by R326 at the end of the S6 helix under acidic conditions. This changed the shape of the S6 helix from bent to straight.

**8. The fact that the VSDs are missing from the simulations suggests that the results presented may not have been observed if the VSDs had been present. In other words, the present simulations may not provide a realistic model of the closed state of the full channel. The authors should comment on that in the manuscript.**

- Thank you for your comment. We modified the following sentence.

**Before the change (in discussion part).**

•The molecular mechanism of the voltage-dependent gating of the Kv channels involves displacement of the voltage-sensing domains that regulate the wetting or dewetting of the cavity [7]. Therefore, the coexistence of these voltage- and acid-dependent gating mechanisms in Kv1.2 channels implies that both the voltage-induced structural pressure and acid-induced structural pressure can simultaneously influence the gating of the channels.

==============================================================================

**After the change (in discussion part).**

• The molecular mechanism of the voltage-dependent gating of the Kv channels involves displacement of the VSD that regulate the wetting or dewetting of the cavity [7]. The previous study shows that the S4 helix is the main moving part in the VSD which moved down ~15Å across the membrane during the deactivation, and the motion of S4 affects the pore domain through the S4-S5 linker [7]. In our simulation, the intra-subunit interaction between K312 and E420 is affected by the protonation state of E420, so the K312 (locate at the S4-S5 linker) could restrict the motion of S4 depending on the environmental pH condition. As a result, S4-S5 linker is affected by environmental pH. Since we simulated with the pore domain (residues 312–421, without VSDs) of Kv1.2, it does not fully reflect the realistic motion of a full chain of Kv1.2 with VSDs. However, it would be used to understand the effect of the acidic environment on channel gating of the pore domain. The coexistence of these voltage- and acid-dependent gating mechanisms in Kv1.2 channels implies that both the voltage-induced structural pressure and acid-induced structural pressure can simultaneously influence the gating of the channels.

**9. Correlation coefficients should be provided for the left panels of Fig. 5.**

-Thank you for your comment, we modified Figure 5.

**10. Methods: Why the very high 0.6 M salt concentration? What did the “20,000 steps of equilibrating simulations” consist of?**

-In the preliminary experimental studies, we confirmed the permeation of K+ ions according to the applied voltage. Frequent observation of ion permeations required high ion concentrations. Additionally, we wanted to compare the result with previous simulation studies performed with 0.6M ion concentration. *(ref. : Jensen MØ, Jogini V, Borhani DW, Leffler AE, Dror RO, Shaw DE. Mechanism of voltage gating in potassium channels. Science. 2012;336(6078):229-33, Jensen MO, Borhani DW, Lindorff-Larsen K, Maragakis P, Jogini V, Eastwood MP, et al. Principles of conduction and hydrophobic gating in K+ channels. Proc Natl Acad Sci U S A. 2010;107(13):5833-8.)*

We performed the heating process by gradually removing the restraint on lipids and protein over 20,000 steps for the stable 310K system temperature.

We modified the following sentence.

**Before the change (in methods part).**

•Approximately 5,000 steps of steepest-descent minimization and 20,000 steps of equilibrating simulation were conducted.

==============================================================================

**After the change (in methods part).**

•Approximately 5,000 steps of steepest-descent minimization were conducted. We performed the heating process by gradually removing the restraint on lipids and protein over 20,000 steps for the stable 310K system temperature.

**11. “After applying MEAD programs” is vague. Enough details should be provided on the pKa calculations for a competent researcher to be able to reproduce the results provided.**

-We inserted more detailed part of MEAD programs as following

**Before the change (in methods part).**

•For pKa estimates of H418 and the acidic residues in the pore domain of the closed Kv1.2 channels, we extracted 98 ensemble structures in the last 1-μs time window from our MD simulation for a Ep327/Hp418/Ep420 state. In addition, after applying MEAD programs [15], we selected 765 titration states of the channels that were predominant in the pH range from 3 to 8. At each titration state, we calculated the energy for each of 98 ensemble structures, as well as their average and standard deviation using AMBER force field 99SB [31]. Red circles in the figure represent the pKa values when the average energies of ensemble structures, $\left\langle E \right\rangle$, were used to calculate the titration fraction as a function of pH for the corresponding titration states. titration states. ==============================================================================

**After the change (in methods part).**

•For pKa estimates of H418 and the acidic residues in the pore domain of the closed Kv1.2 channels, several processes were executed. 1) We extracted 98 ensemble structures of the Kv1.2 channels with pore closure in the last 1 μs time window from our MD simulation for an Ep327/Hp418/Ep420 state. In our setting of MD simulation the pore domain of Kv1.2 channel together with explicit membrane lipids, water molecules, and ions were included. 2) In order to select preferred titration states among all possible titration states in the pH range from 3 to 8, we took advantage of MEAD algorithms [15] to the 10 out of 98 pore closure ensembles with these options (dielectric constants of a molecular interior region / solvent region were $\varepsilon_{\text{in}}=6.0, \varepsilon_{\text{sol}}=80.0$, ionic strength was 0.15 mol/l, and the effect of membrane was excluded.). 3) We selected 765 titration states of the channels that were predominant in the pH range from 3 to 8. For the estimation of pKa values we utilized these 765 titration states and 10 pore closure structural ensembles from our MD simulation. 4) At each titration state on all 98 ensemble structures, we calculated the system energies in the implicit water using AMBER force field 99SB [31]. 5) From the titration states of 3) and the system energies of 4), we reconstructed the partition function, the protonated fractions and estimated pKa values of H418 and the acidic residues (Fig 2A) [33]. Red circles in the figure represent the pKa values when the average energies (using AMBER force field 99SB) of ensemble structures, $\left\langle E \right\rangle$, were used to calculate the protonation fraction as a function of pH for the corresponding titration states.

**12. In the description of pKa estimates, “uncertainty” should be used rather than “flexibility”.**

-We modified to the recommended word as following

**Before the change (in methods part).**

•The blue crosses represent the pK_a_ values when the statistical variance of the energies $\left\langle E \right\rangle\pm0.1\sigma, \left\langle E \right\rangle\pm0.2\sigma, \ldots,\left\langle E \right\rangle\pm1\sigma$ was considered to reflect the flexibility of the pK_a_ values due to the structural fluctuation of ensemble structures.

==============================================================================

**After the change (in methods part).**

•The blue crosses represent the pK_a_ values when the statistical variance of the energies $\left\langle E \right\rangle\pm0.1\sigma, \left\langle E \right\rangle\pm0.2\sigma, \ldots,\left\langle E \right\rangle\pm1\sigma$ was considered to reflect the uncertainty of the pK_a_ values due to the structural fluctuation of ensemble structures.
