# Supplementary material for: Structural and molecular insight into the pH-induced low-permeability of the voltage-gated potassium channel Kv1.2 through dewetting of the water cavity
Source: PLoS Comput Biol. 2020 Apr 21;16(4):e1007405. doi: 10.1371/journal.pcbi.1007405 (PMC7173763; doi:10.1371/journal.pcbi.1007405)
Supplement: Attachment — Submitted filename: Respons_2020_03_04.docx [file pcbi.1007405.s011.docx]

Reviewer #3: Some of my earlier concerns have not been addressed satisfactorily.

**1. In particular, my earlier point #3: To support the conclusions of the paper, it is essential to report the full detailed analysis of all 8 systems studied in order to provide controls for the proposed mechanism. To address this request, the authors have added supplementary figures showing full analysis of the other systems. However, they do not mention or discuss these results explicitly in the main text. This is all the more surprising because the results actually corroborate their mechanistic conclusions regarding the effect of charge-charge interactions on the structure and gating of the pore domain. The authors should discuss these results, which would significantly strengthen the paper.**

- We didn’t mean neglecting your suggestion. We agreed with your point, and now modify the following sentence to express your point in the main text of manuscript. In the revised manuscript we mentioned that our MD simulations for all eight variants showed the structural transition from the open to the closed conformation for at least 1 out of 5 trajectories with the same molecular mechanism of the pore closure of Ep327/Hp418 state.

We modified the following sentence.

**Before the change (in main part).**

• The conformation of the Kv1.2 channel was further probed by examining the changes in the inter-subunit interactions among R326, E327, and H418 and the intra-subunit interaction between K312 and E420. We were able to modify the charge states of these residues through protonation or mutation, as shown in Table 1. First, we modified the inter-subunit interaction by changing the charge state of H418 to resemble the weak acidic condition (Fig S2 and S6). Second, we changed the charge states of both E327 and H418 to break the inter-subunit interaction, which resembled the stronger acidic condition (Fig S3 and S7). Third, we modified the intra-subunit interaction by changing the charge state of E420 (Fig S4 and S8). Finally, we broke both the inter- and intra-subunit interactions by simultaneously changing the charge state of E327, H418, and E420, which resembled the strong acidic condition (Fig S5 and S9).

==============================================================================

**After the change (in main part).**

• The conformation of the Kv1.2 channel was further probed by examining the changes in the inter-subunit interactions among R326, E327, and H418 and the intra-subunit interaction between K312 and E420. We were able to modify the charge states of these residues through protonation or mutation. First, we modified the inter-subunit interaction by changing the charge state of H418 to Hp418 (Fig S2) or H418R (Fig S6) which resembled the weak acidic condition. Second, we broke the inter-subunit interactions by changing to Ep327/Hp418 (Fig S3) or E327A/H418R (Fig S7) which resembled the strong acidic condition. Third, we modified the intra-subunit interaction by changing the charge state of E420 to Ep420 (Fig S4) or E420A (Fig S8). The protonation or mutation of E420 weakens the charge interaction between E420 and K312. It destabilized the open conformation of the Kv1.2 channel and induced conformational change from open to closed. Additionally, these effect on K312, which located in the S4-S5 linker, could propagate to the VSD throughout the S4 helix. Finally, we broke both the inter- and intra-subunit interactions by changing to Ep327/Hp418/Ep420 (Fig S5) or E327A/H418R/E420A (Fig S9) which resembled stronger acidic condition.

Our MD simulations for all 8 variants showed the structural transition from the open to the closed conformation in the pore of the Kv1.2 channel for at least 1 out of 5 trajectories (Fig S2E to S9E). The closed conformations of the variants commonly showed both an increase in the inter-subunit distance (Fig S2C to S9C) and a straightening of the S6 helix (Fig S2D to S9D), as like as the molecular mechanism of the pore closure of Ep327/Hp418 state. Of the variants, the correlation values between the R326-H418 distance and the dihedral angles range from 0.64 to 0.89 (Fig S2A to S9A), and the correlation values between the number of cavity water and the dihedral angles range from 0.49 to 0.78 (Fig S2B to S9B). We were thus able to control the pore closure of Kv1.2 through the various protonation or mutation, which corroborated the molecular mechanism regarding the effect of charge-charge interactions on the structure and gating of the pore domain of Kv1.2 channel.

**2. All the responses given to the reviewers should also be reflected in the text. However, this was not done for my previous request #10: the authors should explain the choice of 0.6 M salt concentration not only in their reply to me, but also in the text of the manuscript. This comment also applies to some of the responses made to the other 2 reviewers.**

- Thank you for finding our mistake in the previous revision.

We modified the following sentence.

**Before the change (in method part).**

• The system consists of a channel protein, lipids (271 POPE in upper and lower leaflets), water molecules (~10,000 TIP3P water molecules), and ions (0.6 M; K^+^, 133; Cl^−^, 125; Wild^UnP^ condition).

==============================================================================

**After the change (in method part).**

• The system consists of a channel protein, lipids (271 POPE in upper and lower leaflets), water molecules (~10,000 TIP3P water molecules). In order to conduct the simulation under the same ionic conditions in the previous studies [7, 19], we added 0.6M of K^+^ and Cl^-^ ions.

**Problems with the interpretation of the results:

3. Since none of the closing simulations are at equilibrium, the quantities reported in the Figures as “free energy landscapes (in arbitrary units)” are wrong on two counts: (a) these are not free energies; and (b) free energy is not unitless. I would recommend that the authors report the results as 2D histograms to avoid these issues.**

- We agree with your concern and recommendation about the presentation of “free energies” requiring at equilibrium. Thus, we replaced the presentation of the “free energy landscapes (in arbitrary units)” to “ensembles population of log scale”.

With heat map (Fig 4D, 4E), we not only compared Wild^UnP^ and Ep327/Hp418 but also explained the correlation between R326-H418 distance (number of water molecules in the water cavity) and straightening of H6. Following your suggestion, we added 2D histograms to the heat map as we thought that it would be good to show such correlation more vividly(Fig 4 and Fig S1 to S9).

We modified the following sentence.

**Before the change (in figure legend).**

• Free energy landscapes (in arbitrary units) for the R326–H418 distances and dihedral angles, and **(B)** free energy landscapes (in arbitrary units) for the dihedral angles and number of water molecules in the water-filled cavity.

==============================================================================

**After the change (in figure legend).**

• Ensembles population of log scale for the R326–H418 distances and dihedral angles, and **(B)** ensembles population of log scale for the dihedral angles and number of water molecules in the water-filled cavity.

**Before the change (Fig 4D and 4E). After the change (Fig 4D and 4E).**

We modified the following figures.

**4. In addition, since none of the closing simulations are at equilibrium (observed dewetting transitions being irreversible), the amount of time spent in the closed state (reported as number of snapshots in Table 1) is meaningless. Whether or not closing occurred early or late in any simulation is essentially random (as it would be in an exponential relaxation process). As such, it is more appropriate to report, as the authors did in the previous version of the paper, the number of simulations that underwent irreversible closing events (i.e., at least 1 out of 5 for all the systems except the unprotonated WT), as an indication of the capacity of the systems to undergo dewetting. While all the systems other than the unprotonated WT underwent closing, based on the data provided and the above considerations, it is not possible to quantify the likelihood that any of these systems would close. As such, it is not possible to rank them either. In other words, ALL THE VARIANTS PRODUCED INDISTINGUISHABLE RESULTS. This point should be conveyed in the text.**

- We greatly appreciated your interpretation and agreed with your point. Following your suggestions, we changed the expression for the number of closed conformations to “at least 1 out of 5 trajectories” in the main text of manuscript. Also we removed Table 1, Figure 5 and related expressions (quantitative comparisons between the eight variants are also removed), and emphasized the common mechanism uncovering in the closed conformations of the variants.

We modified the following sentence.

**Before the change (in main part).**

• The number of water molecules in the cavity implicates that the closed conformations were more frequently sampled in Ep327/Hp418 (2915 snapshot) compared to those of Wild^UnP^ (0 snapshot) in Table 1 [17].

• We compared how inter-subunit interaction (R326, E327, and H418) and intra-subunit interactions (K312 and E420) affect the channel gating (Fig 4A). As a result, the closed structures were more frequently observed when changing the inter-subunit interaction (Hp418: 1287 snapshots in Table 1) compared to those of changing the intra-subunit interaction (Ep420: 747 snapshots in Table 1).

**Before the change (in discussion part).**

• We tracked the step-by-step conformational change using MD simulation. Additionally, our mutant studies showed that the inter-subunit interaction (R326, E327, and H418) is more important than the intra-subunit interaction (K312 and E420). Channel gating is controlled by the charge states of the inter-subunit interaction rather than the intra-subunit interaction. This MD simulation result indicating that E327 and H418 are important for channel gating is consistent with our pKa estimate.

• It imply that H418R and E327A/H418R mutants have low ion permeability, even under the neutral pH condition.

==============================================================================

**After the change (in main part).**

• The number of water molecules in the cavity implicates that the closed conformations were sampled in Ep327/Hp418 (Fig 3B, S3), but not in Willd^UnP^ [17].

• We studied how inter-subunit interaction (R326, E327, and H418) affect the channel gating (Fig 4A).

**After the change (in discussion part).**

•We tracked the step-by-step conformational change using MD simulation.

•It imply that H418R and E327A/H418R mutants have low ion permeability, even under the neutral pH condition. With various protonated or mutated MD simulations, we found that the charge interaction of inter- and intra-subunit affects the channel gating. However, because of the sampling issues, we are not sure about a quantitative comparison that which interactions are more important. Therefore, additional in-vivo or in-vitro experiments are needed for the quantitative comparison.

We modified sentences in the subtitle **"Altering the conformation of Kv1.2 through mutation of E327 and H418"**.

**Before the change (in “Altering the conformation of Kv1.2 through mutation of E327 and H418”)**

• The conformation of the Kv1.2 channel was further probed by examining the changes in the inter-subunit interactions among R326, E327, and H418 and the intra-subunit interaction between K312 and E420. We were able to modify the charge states of these residues through protonation or mutation, as shown in Table 1. First, we modified the inter-subunit interaction by changing the charge state of H418 to resemble the weak acidic condition (Fig S2 and S6). Second, we changed the charge states of both E327 and H418 to break the inter-subunit interaction, which resembled the stronger acidic condition (Fig S3 and S7). Third, we modified the intra-subunit interaction by changing the charge state of E420 (Fig S4 and S8). Finally, we broke both the inter- and intra-subunit interactions by simultaneously changing the charge state of E327, H418, and E420, which resembled the strong acidic condition (Fig S5 and S9). Our simulation results showed that the transition from the open to the closed conformation in the pore of the Kv1.2 channel depends on the state of the inter-subunit interaction (Table 1). The E327 and H418 residues located on the intracellular side across the membrane have a major effect on the pH-sensing mechanism, depending on their protonation states.

E327A/H418R mutant mimics the effect of acidic pH conditions. As expected, our simulation results showed that the closed conformations were more frequently sampled in E327A/H418R mutant (1807 snapshots) compared to those of the Wild^UnP^ (0 snapshots) in Table 1. In addition, the molecular mechanism of the pore closure of the mutant was similar to that of the Ep327/Hp418 state of Kv1.2, showing both an increase in the inter-subunit distance and a straightening of the S6 helix (right panels of Figs 5A, 5B, S6, and S7). For the pKa estimation of each titratable acidic residue in each subunit of the Kv1.2 channel, all four H418 residues in the four subunits were protonated under acidic pH conditions, whereas only two E327 residues in the four subunits were protonated (Fig 2A). Thus, we believe that the H418 residue is a more suitable target of a single mutation than E327. As expected, our simulation results showed that the closed conformations were more frequently sampled in H418R mutant (1835 snapshot) compared to those of the Wild^UnP^ (0 snapshot) in Table 1. In the left panels of Figs 5A and 5B, the closed conformation of the H418R mutant almost resembled the closed conformations of the Ep327/Hp418 state and the E327A/H418R mutant. Based on the molecular mechanism of the Kv1.2 channel, we were thus able to control the pore closure of Kv1.2 through mutation of the E327 and/or H418 residues.

**After the change (in “Altering the conformation of Kv1.2 through protonation or mutation”)**

• The conformation of the Kv1.2 channel was further probed by examining the changes in the inter-subunit interactions among R326, E327, and H418 and the intra-subunit interaction between K312 and E420. We were able to modify the charge states of these residues through protonation or mutation. First, we modified the inter-subunit interaction by changing the charge state of H418 to Hp418 (Fig S2) or H418R (Fig S6) which resembled the weak acidic condition. Second, we broke the inter-subunit interactions by changing to Ep327/Hp418 (Fig S3) or E327A/H418R (Fig S7) which resembled the strong acidic condition. Third, we modified the intra-subunit interaction by changing the charge state of E420 to Ep420 (Fig S4) or E420A (Fig S8). The protonation or mutation of E420 weakens the charge interaction between E420 and K312. It destabilized the open conformation of the Kv1.2 channel and induced conformational change from open to closed. Additionally, these effect on K312, which located in the S4-S5 linker, could propagate to the VSD throughout the S4 helix. Finally, we broke both the inter- and intra-subunit interactions by changing to Ep327/Hp418/Ep420 (Fig S5) or E327A/H418R/E420A (Fig S9) which resembled stronger acidic condition.

Our MD simulations for these 8 variants showed the structural transition from the open to the closed conformation in the pore of the Kv1.2 channel for at least 1 out of 5 trajectories (Fig S2E to S9E). The closed conformations of the variants commonly showed both an increase in the inter-subunit distance (Fig S2C to S9C) and a straightening of the S6 helix (Fig S2D to S9D), as like as the molecular mechanism of the pore closure of Ep327/Hp418 state. Of the variants, the correlation values between the R326-H418 distance and the dihedral angles range from 0.64 to 0.89 (Fig S2A to S9A), and the correlation values between the number of cavity water and the dihedral angles range from 0.49 to 0.78 (Fig S2B to S9B). We were thus able to control the pore closure of Kv1.2 through the various protonation or mutation, which corroborated the molecular mechanism regarding the effect of charge-charge interactions on the structure and gating of the pore domain of Kv1.2 channel.

**5. The simulations show that the channel closes when the total charge of the titratable cluster changes by +1, +2, or +3. However, the authors confine their analysis and conclusions to the effect of a +2 change, when in fact the same result is observed with +1 and +3 changes, both with protonation and with mutations. This should be conveyed in the text.**

- As you mentioned, the same results observed when the net charges of the titratable cluster are +1 and +3. So, we modified the following sentence.

**Before the change (in figure legend).**

• The inter-subunit interaction, which consists of R326, E327, and H418, is critically destabilized by the change in the charge states of glutamic acid and histidine. Because the net charge of the inter-subunit network is changed from 0 to +2, the repulsive force between R326 and Hp418 causes the distance to increase from 6.5 Å to 22.3 Å. This repulsive force pushes the end of S6, leading to its distortion.

==============================================================================

**After the change (in figure legend).**

• Inter- and intra-subunit interactions, which consists of K312, R326, E327, H418, and E420, were critically destabilized by the change in the charge states of these titratable residues. Because the net charge of this cluster changes from zero to positive (+1, +2, or +3), the intra-subunit interaction K312-Ep420 weakens and the repulsive force between R326 and Hp418 increases the distance. This repulsive force pushes the end of S6, leading to its distortion.

**6. The statement that “the H418 residue is a more suitable target for a single mutation than E327” is not supported by the data shown and should be removed. There is no discernible effect of changing one residue or the other or both.**

- We removed this statement together with modifying the sentences in the fourth comment.

**Before the change (in “Altering the conformation of Kv1.2 through mutation of E327 and H418”)**

• E327A/H418R mutant mimics the effect of acidic pH conditions. As expected, our simulation results showed that the closed conformations were more frequently sampled in E327A/H418R mutant (1807 snapshots) compared to those of the Wild^UnP^ (0 snapshots) in Table 1. In addition, the molecular mechanism of the pore closure of the mutant was similar to that of the Ep327/Hp418 state of Kv1.2, showing both an increase in the inter-subunit distance and a straightening of the S6 helix (right panels of Figs 5A, 5B, S6, and S7). For the pKa estimation of each titratable acidic residue in each subunit of the Kv1.2 channel, all four H418 residues in the four subunits were protonated under acidic pH conditions, whereas only two E327 residues in the four subunits were protonated (Fig 2A). Thus, we believe that the H418 residue is a more suitable target of a single mutation than E327. As expected, our simulation results showed that the closed conformations were more frequently sampled in H418R mutant (1835 snapshot) compared to those of the Wild^UnP^ (0 snapshot) in Table 1. In the left panels of Figs 5A and 5B, the closed conformation of the H418R mutant almost resembled the closed conformations of the Ep327/Hp418 state and the E327A/H418R mutant. Based on the molecular mechanism of the Kv1.2 channel, we were thus able to control the pore closure of Kv1.2 through mutation of the E327 and/or H418 residues.

==============================================================================

**After the change (in “Altering the conformation of Kv1.2 through protonation or mutation”)**

• Our MD simulations for all 8 variants showed the structural transition from the open to the closed conformation in the pore of the Kv1.2 channel for at least 1 out of 5 trajectories (Fig S2E to S9E). The closed conformations of these 8 variants commonly showed both an increase in the inter-subunit distance (Fig S2C to S9C) and a straightening of the S6 helix (Fig S2D to S9D), as like as the molecular mechanism of the pore closure of Ep327/Hp418 state. The correlation values in the heat maps for the R326-H418 distance and dihedral angles range from 0.64 to 0.89 (Fig S2A to S9A), and the correlation values in the heat maps for the number of cavity water and dihedral angles range from 0.49 to 0.78 (Fig S2B to S9B). We were thus able to control the pore closure of Kv1.2 through the various protonation or mutation, which corroborated the molecular mechanism regarding the effect of charge-charge interactions on the structure and gating of the pore domain of Kv1.2 channel.
